# Characterization of multiple soluble immune checkpoints in individuals with different *Mycobacterium tuberculosis* infection status and dynamic changes during anti-tuberculosis treatment

**DOI:** 10.1186/s12879-022-07506-z

**Published:** 2022-06-14

**Authors:** Huaxin Chen, Jingyu Zhou, Xinguo Zhao, Qianqian Liu, Lingyun Shao, Yehan Zhu, Qinfang Ou

**Affiliations:** 1 Department of Pulmonary and Critical Care Medicine, The First Affiliated Hospital of Soochow University, 899 Pinghai Road, Suzhou, 215006 China; 2 Department of Infectious Diseases, Shanghai Key Laboratory of Infectious Diseases and Biosafety Emergency Response, National Medical Center for Infectious Diseases, Huashan Hospital, Fudan University, Shanghai, 200040 China; 3 Department of Pulmonary Diseases, Wuxi Infectious Diseases Hospital, 1215 Guangrui Road, Wuxi, 214005 China

**Keywords:** Soluble, Immune checkpoint, *Mycobacterium tuberculosis*, Tuberculosis, Infection, Tuberculous pleurisy, Treatment, BTLA

## Abstract

**Background:**

Immune checkpoints are crucial for the maintenance of subtle balance between self-tolerance and effector immune responses, but the role of soluble immune checkpoints (sICs) in *Mycobacterium tuberculosis* (*M. tb*) infection remains unknown. We assessed the levels of multiple sICs in individuals with distinct *M. tb* infection status, and their dynamic changes during anti-tuberculosis treatment.

**Methods:**

We enrolled 24 patients with pulmonary tuberculosis, among which 10 patients were diagnosed with tuberculous pleurisy (TBP), 10 individuals with latent tuberculosis infection (LTBI), and 10 healthy volunteers from Wuxi Fifth People’s Hospital and Huashan Hospital between February 2019 and May 2021. Plasma concentrations of thirteen sICs were measured at enrollment and during anti-tuberculosis treatment using luminex-based multiplex assay. sICs levels in tuberculous pleural effusion (TPE) and their relations to laboratory test markers of TPE were also assessed in TBP patients.

**Results:**

The circulating levels of sPD-1, sPD-L1, sCTLA-4, sBTLA, sGITR, sIDO, sCD28, sCD27 and s4-1BB were upregulated in tuberculosis patients than in healthy controls. A lower sPD-L1 level was found in LTBI individuals than in tuberculosis patients. In TBP patients, the levels of sPD-1, sPD-L2, sCD28, sCD80, sCD27, sTIM-3, sLAG-3, sBTLA, s4-1BB and sIDO increased significantly in TPE than in plasma. In TPE, sBTLA and sLAG-3 correlated positively with the adenosine deaminase level. sIDO and sCD80 correlated positively with the lactate dehydrogenase level and the percentage of lymphocytes in TPE, respectively. Meanwhile, sCD27 correlated negatively with the specific gravity and protein level in TPE. In tuberculosis patients, the circulating levels of sBTLA and sPD-L1 gradually declined during anti-tuberculosis treatment.

**Conclusions:**

We characterized the changing balance of sICs in *M. tb* infection. And our results revealed the relations of sICs to laboratory test markers and treatment responses in tuberculosis patients, indicating that certain sICs may serve as potential biomarkers for disease surveillance and prognosis of tuberculosis.

**Supplementary Information:**

The online version contains supplementary material available at 10.1186/s12879-022-07506-z.

## Background

Tuberculosis (TB) is a communicable disease caused by *Mycobacterium tuberculosis* (*M. tb*), which infected about a quarter of world’s population [1]. Until the pandemic of COVID-19, TB remains the leading cause of death worldwide from a single infectious pathogen. In 2020, approximately 5.8 million people were newly diagnosed with TB, among which over 157 thousand were detected with drug-resistant TB [1]. The continuous high disease burden worldwide urges the development of new strategies for TB treatment and prevention. Host-directed therapy is one of the promising approaches, given the outcome of human infection with *M. tb* is largely dependent on host immune status. Most people infected with *M. tb* can be asymptomatic during their lifetime, with only about 5% fail to contain *M. tb* and eventually develop TB [2]. Research into the interplay between host immune system and pathogen is vital for the development of novel host-directed therapy for TB.

Immune checkpoint proteins can regulate the immune response in malignancies and infectious diseases via numerous types of activating and inhibitory signals between antigen-presenting cells (APCs) and T cells [3, 4]. Receptor-ligand interaction is required for the transduction of second signal, following the first signal conveyed by the interaction of MHC molecules on APCs and T cell receptors on effector T cells loaded with cognate antigens [3]. Co-stimulatory receptor-ligand interactions that help amplify effector T cell responses include CD28–CD80, 4-1BB (also known as CD137)-4-1BB ligand, CD27–CD70. To avoid over-reactivation, effector T cell response also require suppression signals generated upon co-inhibitory receptor–ligand interactions, including B- and T-lymphocyte attenuator (BTLA)-herpes virus entry mediator (HVEM), glucocorticoid-induced TNFR-related (GITR)- GITR ligand, lymphocyte-activation gene 3 (LAG-3)- MHC, programmed cell death protein 1 (PD-1)- programmed cell death 1 ligand 1 (PD-L1)/programmed cell death 1 ligand 2 (PD-L2), T-cell immunoglobulin and mucin domain-3 (TIM-3)- Galectin 9, cytotoxic T-lymphocyte associated antigen 4 (CTLA-4)- CD80. Intracellular molecules, such as indoleamine 2,3-dioxygenase (IDO) were also found to exert immune checkpoint functions in T cells [3]. Researches have investigated the impact of immune checkpoints on the immune responses against a range of chronic infections, including malaria, hepatitis B virus and human immunodeficiency virus infection [3, 5]. Since a well-balanced host immune response against *M. tb* is required to clear or contain the infection, while preventing potentially damaging inflammatory response [6], further insight into the role of immune checkpoints in anti-TB immunity is required for the development of immune therapy for TB.

In this study, we aimed to provide preliminary data on the characteristic profile of soluble immune checkpoints (sICs) in patients with distinct *M. tb* infection status, and their association with immune response at lesion sites, and to assess their dynamic change during anti-TB treatment.

## Methods

### Study population

In total, 44 individuals were enrolled in this study, including 10 individuals with latent tuberculosis infection (LTBI), and 10 healthy controls (HC), and 24 patients with pulmonary tuberculosis (PTB), among which 10 patients were diagnosed with tuberculous pleurisy (TBP). All the TB patients were recruited from Wuxi Fifth People’s Hospital between February 2019 and May 2021. Individuals with LTBI and HC were recruited from the relatives of TB patients and the volunteers of Huashan Hospital during the same period.

PTB patients were diagnosed based on chest radiological evidence, supplemented by a positive result of culture for *M. tb* and/or Xpert MTB/RIF test in the sputum or bronchoalveolar lavage fluid; and individuals clinically diagnosed based on chest radiological evidence, supplemented by clinical symptoms of TB, or a positive tuberculin skin test or interferon-γ release assay (IGRA) result were also included. Patients with TBP were defined as TB patients showed chest radiological evidence of pleural involvement, and met one of the following criteria: a positive result of culture for *M. tb* and/or Xpert MTB/RIF test in the pleural effusion; histologically confirmed *M. tb* infection by pleural biopsy; or an elevated concentration of adenosine deaminase (ADA) in pleural effusion along with a positive tuberculin skin test or IGRA result. Individuals with LTBI and HC were IGRA-positive and -negative, respectively. In addition, they had no evidence of active tuberculosis (ATB). All enrolled participants were free of HIV infection, autoimmune disease or other chronic infections, and not undergoing immune-modulating treatment.

Peripheral blood was drawn from all participants, while pleural effusion was only collected from TBP patients. Plasma and tuberculous pleural effusion (TPE) supernatant were separated and stored at – 80 °C until further assessment. Demographic and clinical characteristics, including age, gender, bacillus Calmette-Guerin (BCG) vaccination history, results of diagnostic tests for TB, and laboratory test results of TPE were also collected.

The study was approved by the institutional ethics review board of Huashan Hospital, Fudan University. Verbal informed consent was obtained for all the investigations, and written informed consent was obtained from each participant in the study prior to enrollment. The study was performed in accordance with the guidelines of the Declaration of Helsinki and relevant regulations.

### Quantification of sICs

Plasma samples of PTB and TBP patients were collected at baseline prior to anti-TB treatment and during treatment. Baseline TPE samples were additionally collected in TBP patients. The ProcartaPlex™ Human Immuno-Oncology Checkpoint Panel Immunoassay Kit (Thermo Fisher, Waltham, MA) was used to measure the concentrations of fourteen sICs, including soluble BTLA (sBTLA), sHVEM, sGITR, sIDO, sLAG-3, sPD-1, sPD-L1, sPD-L2, sTIM-3, sCD28, sCD80, s4-1BB, sCD27 and sCTLA-4. According to the manufacturer’s protocol, all samples were assayed in duplicate, using 25 μL of sample per well. The concentration of the samples was calculated by plotting the expected concentration of the standards against the fluorescent signals generated by each standard. Detection and analysis of assayed samples were performed using the Luminex 200™ System (Thermo Fisher, Waltham, MA, USA).

### Statistical analysis

Statistical analysis was performed using GraphPad Prism 8 (GraphPad Software, Inc. La Jolla, CA, USA). Continuous variables were compared between independent groups using Mann–Whitney test (two groups) and Kruskal–Wallis test (multiple groups). Categorical variables were compared using Fisher's exact test. Paired data were analyzed using the paired Wilcoxon rank test. The correlation between laboratory test markers of pleural effusion, including specific gravity, protein, ADA, lactate dehydrogenase (LDH) and lymphocyte percentage, and the levels of sICs was assessed with Spearman correlation. The results with a *P* value of < 0.05 were considered significant.

## Results

### Clinical characteristics of participants

The 44 enrolled participants were divided into four groups, as shown in Table 1. The PTB group included 14 PTB patients without pleural effusion, which were consist of 6 cases with a positive result of culture for *M. tb*, 3 cases with a positive result of Xpert MTB/RIF test and 5 cases with clinical diagnosis. The TBP group comprised 10 PTB patients with pleural effusion, among which, 3 cases were confirmed by either pleural biopsy (n = 2) or Xpert MTB/RIF of pleural effusions (n = 1), while half (n = 5) scored positive in culture of sputum for *M. tb*. Three participants from the PTB group and two participants from the TBP group had co-morbidities. In this study, all of the patients with ATB were anti-TB treatment naïve at recruitment. The demographic and clinical characteristics of all individuals are presented in Table 1.

**Table 1:** The demographic and clinical characteristics of study participants

Group	ATB			LTBI (n = 10)	HC (n = 10)
	Total (n = 24)	PTB (n = 14)	TBP (n = 10)		
Age, median (IQR)	40.5 (37)	35 (29.25)	49 (49)	50.5 (32.75)	43.5 (26)
Male, n (%)	18 (75)	9 (64.3)	9 (90)	3 (30)	1 (10)
BCG vaccination, n (%)	18 (75)	10 (71.4)	8 (80)	8 (80)	9 (90)
Culture positive, n (%)	11 (45.8)	6 (42.9)	5 (50)	–	–
Xpert MTB/RIF positive, n (%)	4 (16.7)	3 (21.4)	1 (10)	–	–
Confirmed TB by pathology, n (%)	2 (8.3)	–	2 (20)	–	–
Co-morbidity, n (%)					
Diabetes	1 (4.2)	–	1 (10)	–	–
Hypertension	3 (12.5)	2 (14.3)	1 (10)	–	–
Arrhythmia	1 (4.2)	1 (7.1)	–	–	–

*ATB* active tuberculosis, *PTB* pulmonary tuberculosis, *TBP* tuberculous pleurisy, *LTBI* latent tuberculosis infection, *HC* healthy controls, *BCG* bacillus Calmette-Guerin, *TB* tuberculosis

### Circulating sICs levels at baseline in individuals with distinct *M. tb* infection status

**Fig. 1:**
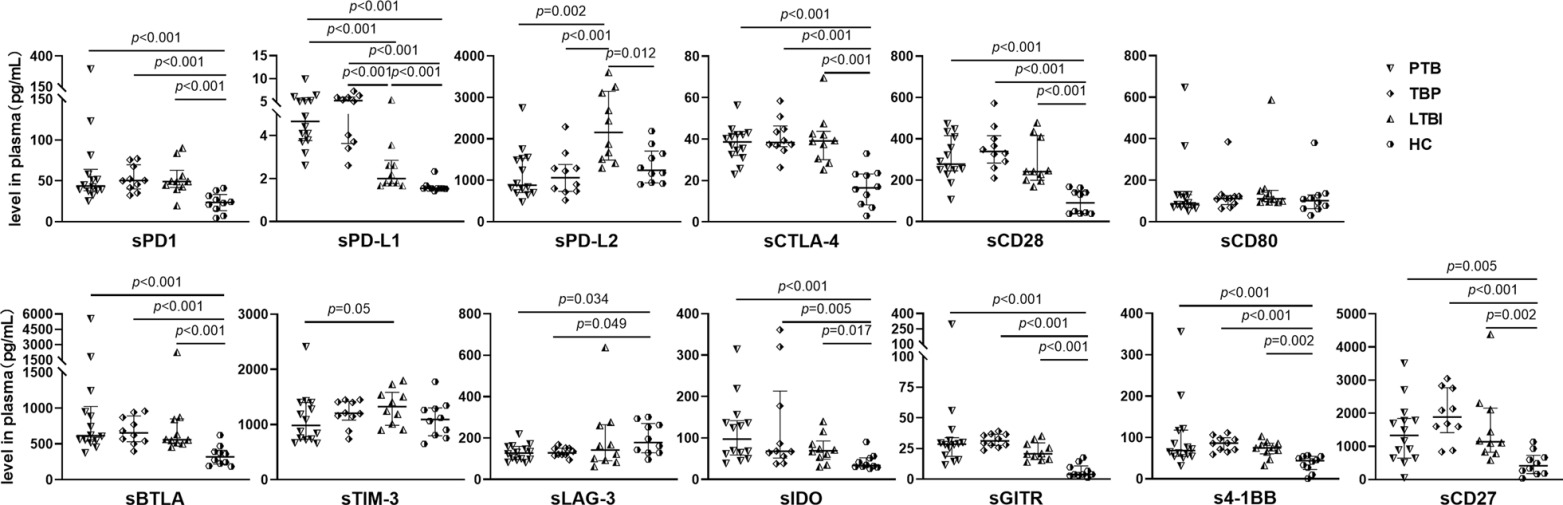
Profile of circulating sICs levels in individuals with distinct *M. tb* infection status. Dot plots of the circulating levels of co-inhibitory sICs, including sPD-1, sPD-L1, sPD-L2, sCTLA-4, sTIM-3, sLAG-3, sBTLA, sGITR, sIDO, and co-stimulatory sICs, including sCD28, sCD80, sCD27, s4-1BB, in PTB and TBP patients, LTBI and HC individuals at enrollment. *sICs* soluble immune checkpoints, *PTB* pulmonary tuberculosis, *TBP* tuberculous pleurisy, *LTBI* latent tuberculosis infection, *HC* healthy controls

A multiplexed fluorescent bead-based immunoassay was performed to determine the baseline circulating sICs levels using plasma of ATB patients prior to anti-TB treatment, individuals with LTBI and HC (Fig. 1). The level of sHVEM was below the limit of detection in almost all samples, thus excluded from subsequent analyses. Compared with the HC group, the concentration of six co-inhibitory sICs, including sPD-1, sPD-L1, sCTLA-4, sBTLA, sGITR and sIDO, as well as three co-stimulatory sICs, including sCD28, sCD27 and s4-1BB, were significantly increased in plasma from participants with PTB, TBP and LTBI, with an exception of sLAG-3, another co-inhibitory sIC, showing a downregulation in participants with *M. tb* infection. Meanwhile, an upregulation of co-inhibitory sPD-L2 in LTBI participants, and a contradictory downregulation of sPD-L2 were simultaneously found in ATB patients. In comparison with the LTBI individuals, a higher level of sPD-L1 were found in both the PTB and TBP group. A trend of decrease of co-inhibitory sTIM-3 level was found in the PTB patients, but the level of sTIM-3 and sCD80 showed no significant difference between the four groups. No significant change of the thirteen sICs in circulation was found between the PTB and TBP group.

### Comparison of sICs levels in plasma and TPE in patients with TBP

**Fig. 2:**
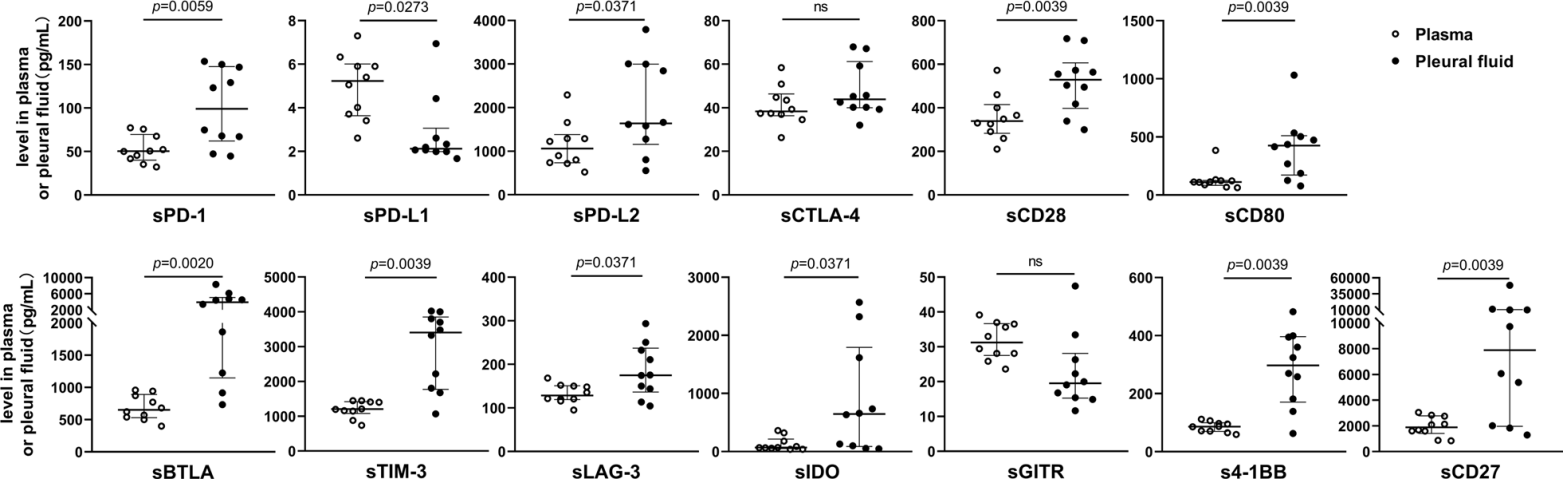
Comparison of sICs levels in plasma and TPE in patients with TBP. Dot plots of sICs levels in TPE and paired plasma from TBP patients at baseline prior to anti-TB treatment. *sICs* soluble immune checkpoints, *TPE* tuberculous pleural effusion, *TBP* tuberculous pleurisy, *TB* tuberculosis

The concentrations of sICs were also measured using TPE of patients with TBP collected prior to treatment and compared with those in paired plasma. (Fig. 2) The majority of the sICs, including sPD-1, sPD-L2, sCD28, sCD80, sCD27, sTIM-3, sLAG-3, sBTLA, s4-1BB, sIDO, showed significant increase in TPE than in plasma, except for the co-inhibitory molecule sPD-L1, showing a decrease in TPE. The levels of sCTLA-4 and sGITR were respectively comparable in plasma and TPE.

### Correlation between laboratory test markers and sICs levels in TPE

**Fig. 3:**
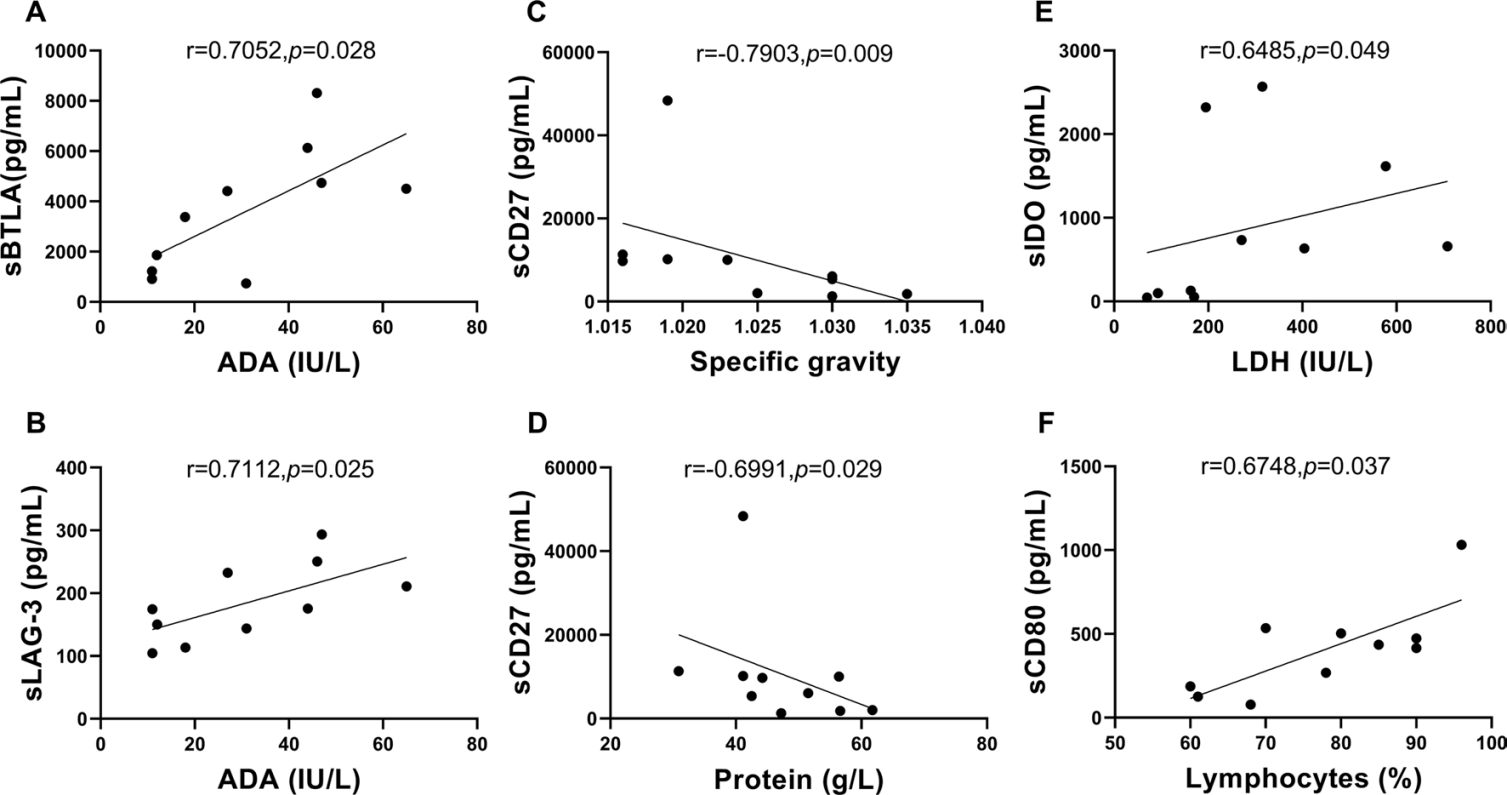
Correlation between laboratory test markers and sICs levels in TPE. Analysis of TPE from TBP patients showed (**A**) sBTLA (r = 0.7052, *P* = *0.028*) and (**B**) sLAG-3 (r = 0.7112, *P* = *0.025*) correlated positively with ADA level, while sCD27 correlated negatively with (**C**) specific gravity (r = − 0.7903, *P* = *0.009*) and (**D**) protein level (r = − 0.6991, *P* = *0.029*). **E** sIDO and (**F**) sCD80 correlated positively with LDH level (r = 0.6485, *P* = *0.049*) and lymphocytes percentage (r = 0.6748, *P* = *0.037*), respectively. *sICs* soluble immune checkpoints, *TPE* tuberculous pleural effusion, *TBP* tuberculous pleurisy, *ADA* adenosine deaminase, *LDH* lactate dehydrogenase

We then analyzed the correlation between laboratory test markers of TPE and sICs levels in TBP patients (Fig. 3). Co-inhibitory proteins sLAG-3 (r = 0.7112, *P* = 0.025) and sBTLA (r = 0.7052, *P* = 0.028) both correlated positively with the ADA level in of TPE (Fig. 3A, B). Meanwhile, the level of co-stimulatory sCD27 in TPE correlated negatively with specific gravity (r = − 0.7903, *P* = 0.009) and protein level (r = − 0.6991, *P* = 0.029) of TPE (Fig. 3C, D). Another co-inhibitory protein sIDO and the co-stimulatory protein sCD80 were found positively correlated with the LDH level (r = 0.6485, *P* = 0.049, Fig. 3E) and the percentage of lymphocytes (r = 0.6748, *P* = 0.037, Fig. 3F) in TPE, respectively.

### Dynamic changes of circulating sICs during anti-TB treatment

**Fig. 4:**
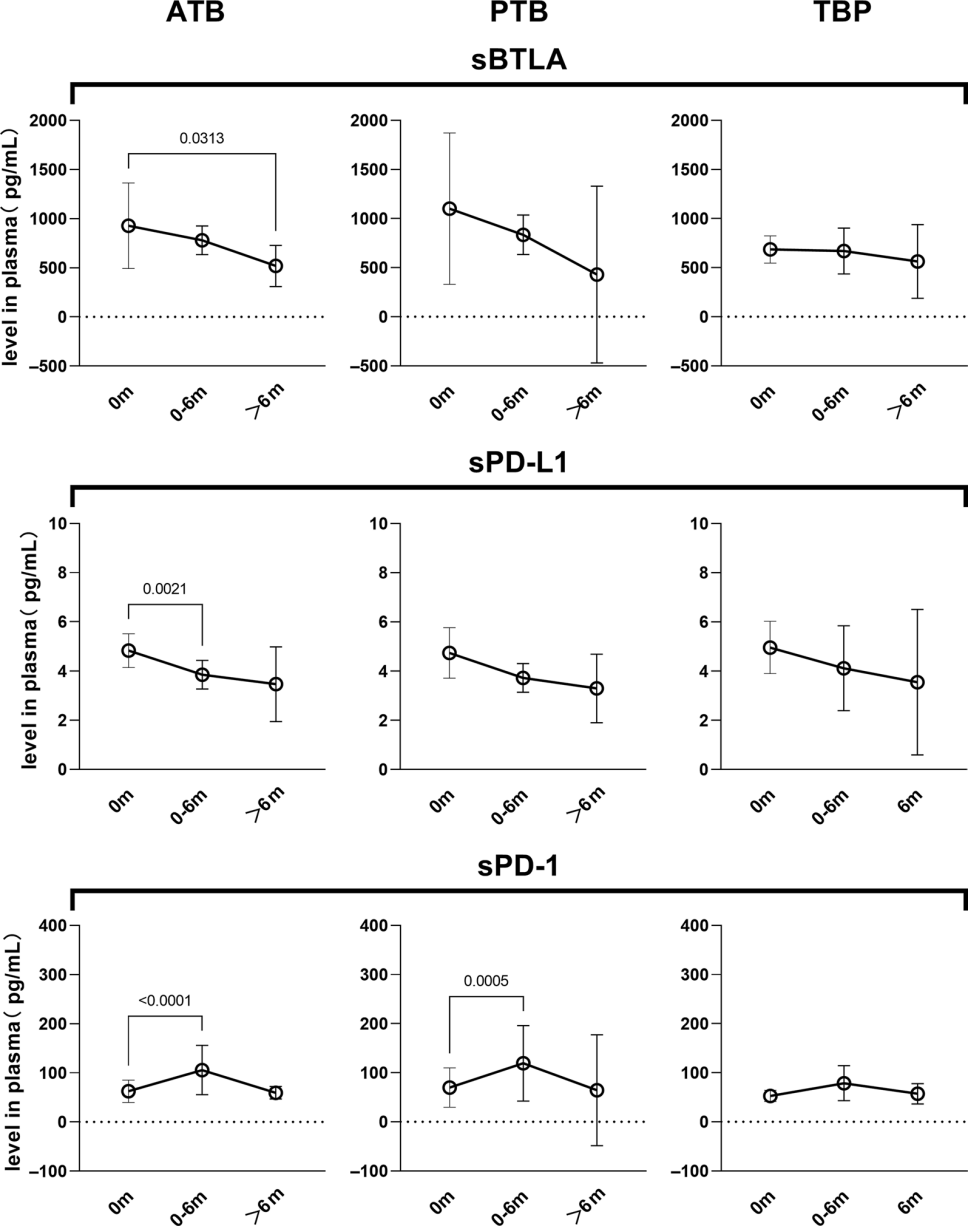
Dynamic changes of circulating sICs during anti-TB treatment. Patients with PTB and TBP were followed up during treatment. The levels of sICs in follow-up plasma were determined and compared with those in paired baseline plasma. The sBTLA level showed a significant decline in ATB patients after 6-month anti-TB treatment (*P* = *0.031*). sPD-L1 showed an early decline in ATB patients (*P* = *0.002*) within 6 months during anti-TB treatment. sPD-1 rose significantly during the 6-month treatment in ATB patients (*P* < *0.001*) and the PTB subgroup (*P* < *0.001*). *sICs* soluble immune checkpoints, *TB* tuberculosis, *PTB* pulmonary tuberculosis, *TBP* tuberculous pleurisy, *ATB* active tuberculosis

Patients with PTB and TBP were followed up during anti-TB treatment, among which 18 were followed up within 6 months and 6 were followed up in 6 or more months after the initiation of anti-TB treatment. The levels of sICs in follow-up plasma were determined and compared with those in paired baseline plasma (Fig. 4). In ATB patients, a significant and continuous decline of sBTLA (*P* = *0.031*) level were found after 6-month anti-TB treatment, while sPD-L1 only showed an early decline within 6 months (*P* = *0.002*). Both sBTLA and sPD-L1 showed similar trends of decline in PTB and TBP patients (*P* > *0.05*). The sPD-1 level of ATB patients (*P* < *0.001*) and the PTB subgroup (*P* < *0.001*) rose significantly during the 6-month treatment and fell back afterward. The levels of the rest 10 sICs showed no significant change during treatment, among which sCTLA-4 (*P* = *0.063*), sCD28 (*P* = *0.063*) and sCD80 (*P* = *0.063*) seemed to decline in ATB patients after 6-month anti-TB treatment, and sTIM-3 (*P* = *0.064*) tended to increase in PTB patients within 6 months (Data shown in Additional file 1). As all the ATB patients responded well to anti-TB treatment and were cured, it can be implied from the results that a sustained downregulation of sBTLA and sPD-L1, rather than the inconsistent changes of sPD-1, might be indicator for preferable anti-TB treatment response.

## Discussion

In this study, we performed comprehensive assessment of the circulating levels multiple sICs in *M. tb* infection for the first time, using four distinct populations, including patients with PTB, TBP, individuals with LTBI, and healthy individuals. We revealed a screwed balance between the co-inhibitory and co-stimulatory molecules in different status of *M. tb* infection.

*M. tb* manipulates host immunity to persist in latent form or develop chronic infection by impairing the bactericidal machinery of APCs and interfering T-cell mediated immunity [7]. In untreated *M. tb* infection, T cells upregulate the display of multiple co-inhibitory molecules, such as PD-1, TIM-3, LAG-3, reduce production of IFN-γ, secrete suppressive cytokines, and proliferate less, suggesting a suppressive immune status in ATB [8]. The expression level of co-inhibitory receptors, PD-1, CTLA-4 and TIM-3, on both lymphocytes and monocytes was also found associated with *M. tb* infection status and changes during LTBI treatment [9]. Similar changes of circulating sICs levels were found in our ATB and LTBI patients. However, such change in plasma sTIM-3 level did not recur in subgroup analysis where culture-negative ATB patients with a clinical diagnosis were excluded from the ATB group, neither did that in plasma sLAG-3 level. One possible explanation could be the correlation between the sICs levels with disease severity, as Wang et al. found the TIM-3 expression on CD8 + T cells in smear-positive PTB patients was significantly higher than that in smear-negative patients [10]. And an elevated sLAG-3 level in PTB patients was also found correlated to with cavity sizes > 4 mm on chest radiography [11].

We have also observed increased sCD28, sCD27 and s4-1BB in *M. tb* infection. Researches showed activated T cells also upregulate numerous co-stimulatory molecules, such as CD28:B7, ICOS:ICOS-L, 4-1BB:4-1BBL, during persisting infections to mount efficient T-cell response [12]. Studies of gene modified murine models and TB patients suggested a positive role of co-stimulatory molecules in anti-TB immunity. A reduced cytokine production and killing capacity of *M. tb*-specific effector CD8 + T cell was found *M. tb*-infected mice with ICOS deficiency during late-stage *M. tb* infection [13]. Together, our results showed a skewed balance of co-inhibitory and co-stimulatory molecules in individuals with ATB and LTBI, suggesting a suppressive immune status in *M. tb* infection.

The sICs levels in plasma and TPE of TBP patients were compared in this study, and most sICs were upregulated, suggesting a more activated and potent anti-TB immune response might exist in pathological lesion sites. The upregulation of co-inhibitory receptor might help prevent overactivated immune response during TBP. As shown in preclinical models, both *M. tb*-infected PD-1 knockout mice and anti-PD-1 antibody-treated rhesus macaques developed detrimental inflammation and exacerbated disease, suggesting checkpoint-mediated co-inhibition is involved in harnessing T-cell-driven immune pathology and the control of *M. tb* infection [14, 15]. There have been several studies on the PD-1/PD-Ls pathways in *M. tb* infection, which all showed an upregulated expression of PD-1 and PD-L1 on both pleural and peripheral CD4 + T cells in ATB patients [16, 17]. Yin et al. also found an increasing sPD-1 level but a comparable level of sPD-L1 level in TPE [17], Partially consistent with the findings of Yin et al., we revealed a higher level of sPD-1 but a significantly lower level of sPD-L1 in TPE, which might result from the repressed release of PD-L1 from pleural mesothelial cells or lymphocytes.

Studies of TBP patients identified the pleural T cells with an effector phenotype of CD27-CD45RA-CCR7-CD62L- mediates local *M. tb*-specific immune response by simultaneously production of multiple cytokines [18, 19]. Consistent with previous finding, we found a low sCD27 in TPE were correlated negatively with the specific gravity and protein level of TPE, which indicates severer chronic inflammation at lesion sites. However, several researches consider the lower expression of CD27 on *M. tb*-specific CD4 + T cells is associated with persistent ATB [20, 21]. Therefore, further investigation on the role of CD27 in *M. tb* infection is needed to draw a conclusion.

LAG-3 expression is significantly induced in the lungs of macaques with ATB, and correlates with diminished responses and increased bacterial burden [22]. Silencing LAG-3 signaling in macaque lung enhanced killing of *M. tb* in CD4 + T cells, by interfering with the mitochondrial apoptosis pathway and increasing IFN-γ expression [23]. We here found that in TPE the sLAG-3 level was positively correlated with the ADA level. As ADA is a highly sensitive and specific marker for TBP diagnosis [24], we surmised sLAG-3 might be a candidate biomarker for TPE.

Follow-up assessment revealed the circulating level of sCTLA4 and sCD28 might be correlated with favorable anti-TB treatment outcome. CTLA4 and CD28 are paired immune checkpoint and co-stimulatory molecules that compete for shared ligands CD80 and CD86 [12]. Studies on chronic viral infection and in vitro blockade showed CTLA4 contribute to the impaired immune response, induction of T cell exhaustion and the failure of immunological control of the persisting pathogens by pathogen-specific T cells [12, 25]. CTLA4 attenuates T cell activation by inhibiting co-stimulatory signal via CD28 and transmitting inhibitory signals to T cells [25]. Our results suggested a recovering balance between co-inhibitory and co-stimulatory signals as the clearance of bacteria load in *M. tb* infection.

More attention should be given to BTLA, since sBTLA was found not only correlated with *M. tb*-specific marker ADA in TPE, but also steadily decline in response to anti-TB treatment. Intense research in the field of cancer, autoimmune diseases and various infection has revealed the interaction of BTLA with its ligand HVEM has a potent inhibitory effect on adaptive, majorly T-cell mediated, immune response [26]. However, the effect and mechanism of BTLA-HVEM signaling in *M. tb* infection still remain poorly understood. Recent studies on *M. tb* infection have found that, in comparison with HC, ATB patients exhibited enhanced expression of BTLA on myeloid and plasmacytoid dendritic cells (DCs) from peripheral blood and TPE, different from the high level of BTLA expression on T cells observed in virus infection [27–29]. TB-driven BTLA upregulation on DCs impaired the expression of DCs mature marker CD83 and activation marker HLA-DR, and suppress the ability of DCs to induce Th17 and Th22 response, while promoting Th2 response [28, 29]. Additionally, BTLA + DCs can promote Foxp3 expression in naïve CD4 + T cells through the upregulation of CD5 and subsequent inhibition of PI3K/mTOR activation, therefore inducing extrathymic Treg differentiation [30]. Together, these findings indicate the activation of BTLA-HVEM pathway is involved in the pathogenesis of TB. Further insight is required to understand the mechanism of BTLA-mediated regulation on anti-TB immunity.

Some limitations in the present study must be noted. First, the number of individuals involved in our study was small. And a number of clinically diagnosed ATB patients who lacks positive bacteriological test results were included. More bacteriologically confirmed ATB patients should be enrolled in further study to provide more power. Second, ATB patients were followed up only once during their anti-TB therapy, resulting from the retrospective study design. Serial follow-up at different time points should be done to depict the kinetics of sICs in a more detailed way. To develop novel biomarkers of TB, further investigation on the sICs that showed correlation with *M. tb* infection status or treatment response in this study is required.

## Conclusions

We here illustrated a skewed balance of co-inhibitory and co-stimulatory molecules exists in human *M. tb* infection. Our results suggested certain immune checkpoint molecules might be involved in the regulation of T-cell mediated immune response, which plays a pivotal part in host adaptive immune against *M. tb*. Upon further study, they could be developed into promising biomarkers for the surveillance and prognosis of TB.

## Supplementary Information

**Additional file 1.** **Supplementary Figure 1.** Profile of baseline circulating sICs levels in definite ATB, LTBI and HC individuals. **Supplementary Figure 2.** Dynamic changes of ten circulating sICs during anti-TB treatment.

## Data Availability

All data generated or analyzed during this study are included in this published article.

## Abbreviations

ADA: Adenosine deaminase
ATB: Active tuberculosis
APCs: Antigen-presenting cells
BCG: Bacillus Calmette-Guerin
DCs: Dendritic cells
HC: Healthy controls
LDH: Lactate dehydrogenase
LTBI: Latent tuberculosis infection
*M. tb*: *Mycobacterium tuberculosis*
PTB: Pulmonary tuberculosis
BTLA: B- and T-lymphocyte attenuator
CTLA-4: Cytotoxic T-lymphocyte associated antigen 4
GITR: Glucocorticoid-induced TNFR-related
HVEM: Herpes virus entry mediator
IDO: Indoleamine 2,3-dioxygenase
LAG-3: Lymphocyte-activation gene 3
PD-1: Programmed cell death protein 1
PD-L1: Programmed cell death-ligand 1
PD-L2: Programmed cell death-ligand 2
sICs: Soluble immune checkpoints
TIM-3: T-cell immunoglobulin and mucin domain-3
TB: Tuberculosis
TBP: Tuberculous pleurisy
TPE: Tuberculous pleural effusion
