# Supplementary material for: Characterization of multiple soluble immune checkpoints in individuals with different Mycobacterium tuberculosis infection status and dynamic changes during anti-tuberculosis treatment
Source: BMC Infect Dis. 2022 Jun 14;22:543. doi: 10.1186/s12879-022-07506-z (PMC9192932; doi:10.1186/s12879-022-07506-z)
Supplement: Supplementary file 1 — Additional file 1. Supplementary Figure 1. Profile of baseline circulating sICs levels in definite ATB, LTBI and HC individuals. Supplementary Figure 2. Dynamic changes of ten circulating sICs during anti-TB treatment. [file 12879_2022_7506_MOESM1_ESM.docx]

**Supplementary materials**

**
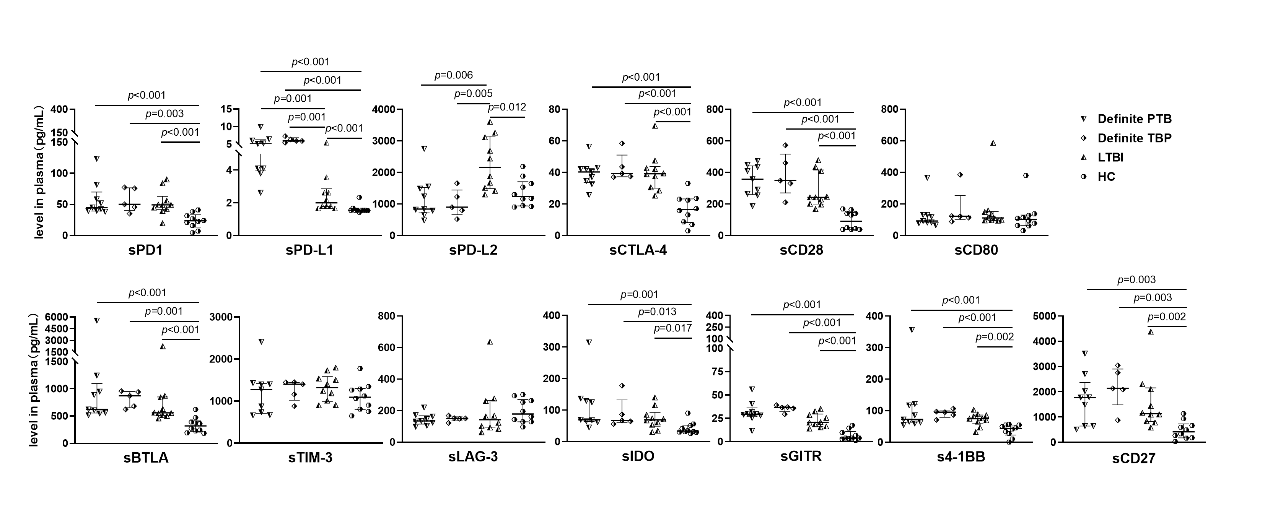
Supplementary Figure 1. Profile of baseline circulating sICs levels in definite ATB, LTBI and HC individuals.**

Dot plots of the circulating levels of sICs in definite PTB and TBP patients, LTBI and HC individuals at enrollment. sICs, soluble immune checkpoints; PTB, Pulmonary tuberculosis; TBP, Tuberculous pleurisy; LTBI, Latent tuberculosis infection; HC, Healthy controls.

Since we included a significant proportion of ATB patients with clinical diagnosis, we built a subgroup of ATB patients with definite diagnosis, comprising 9 PTB and 5 TBP patients, based on a positive culture or Xpert MTB/RIF result. And we reanalyzed the plasma sIC levels between the definite PTB, definite TBP, LTBI and HC group (Figure. S1). Results were similar to those found in analysis of 24 ATB patients with either clinical or definite diagnosis, except for sTIM-3 and sLAG-3. The plasma sTIM-3 and sLAG-3 levels became comparable between all groups. The plasma sPD-1, sCTLA-4, sCD28, sBTLA, sIDO, sGITR, s4-1BB and sCD27 levels remained significantly higher definite ATB patients than HC individuals. And the plasma sPD-L1 level remained significantly lower in definite ATB patients than individuals with LTBI.

**
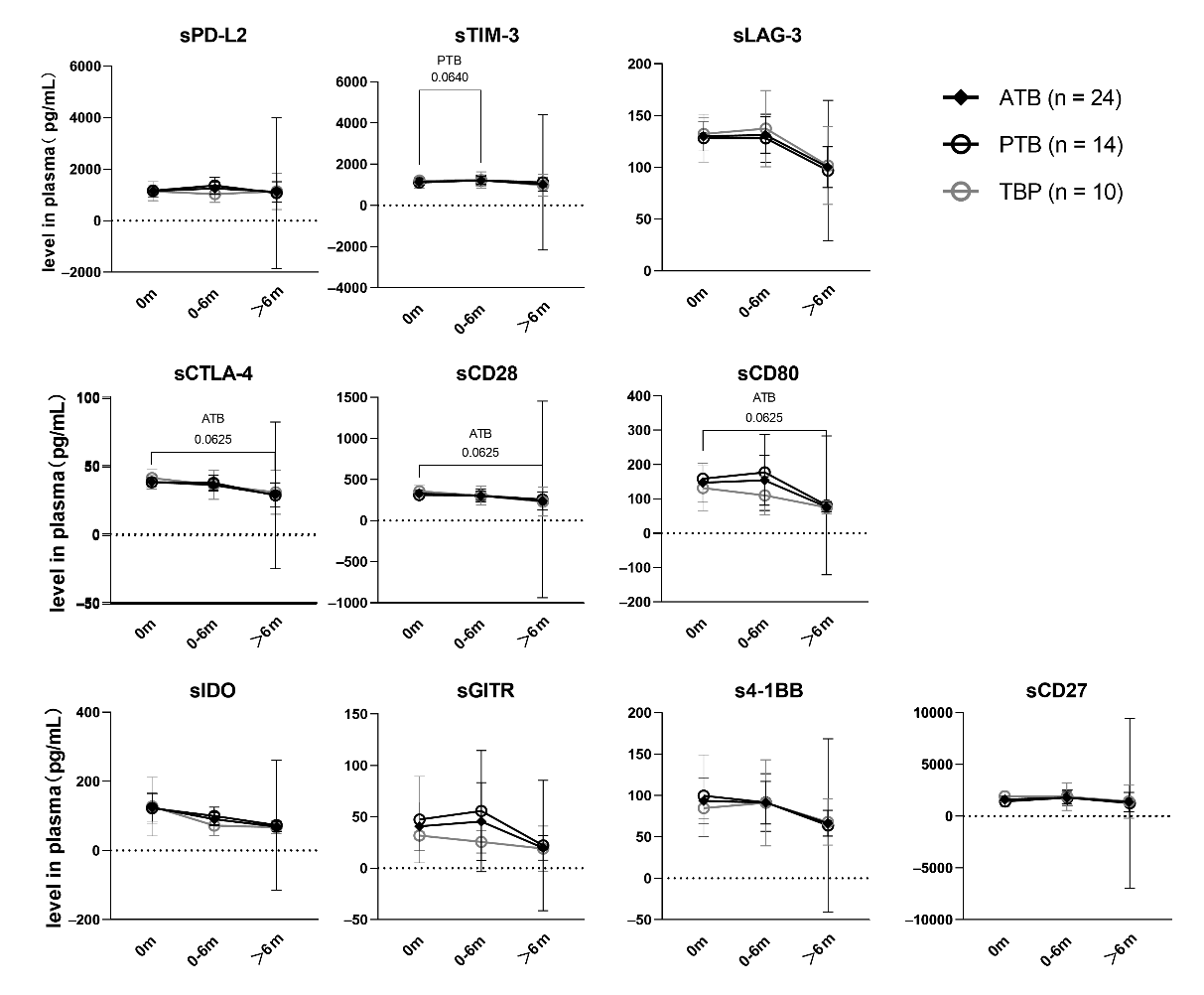
Supplementary Figure 2. Dynamic changes of ten circulating sICs during anti-TB treatment.**

Patients with PTB and TBP were followed up during treatment. The levels of ten sICs in follow-up plasma were determined and compared with those in paired baseline plasma. sICs, soluble immune checkpoints; TB, Tuberculosis; PTB, Pulmonary tuberculosis; TBP, Tuberculous pleurisy; ATB, Active tuberculosis.

The dynamic changes of ten sICs levels among PTB patients (n=14) and TBP patients (n=10) during treatment which were not included in Figure. 4 were shown in Figure. S2. No significant changes among the plasma levels of these sICs during treatment were found. The plasma sCTLA-4 (*P*=0.063), sCD28 (*P*=0.063) and sCD80 (*P*=0.063) levels seemed to decline in ATB patients after 6-month anti-TB treatment. The circulating sTIM-3 level (P=0.064) tended to increase in PTB patients within 6 months after treatment initiation.
